# Genome-Wide Association Mapping and Integrated Transcriptomic-Genomic Analysis Reveal Candidate Genes for Grain Transparency in Rice

**DOI:** 10.3390/ijms27135835

**Published:** 2026-06-28

**Authors:** Fuge Cai, Fujun Wang, Shuai Nie, Minhua Zheng, Song Bai, Hui Li, Meilin Tan, Mengquan Chen, Dilin Liu, Wu Yang

**Affiliations:** 1 Anshun Academy of Agricultural Sciences, Anshun 562100, China; 2 Rice Research Institute, Guangdong Academy of Agricultural Sciences, Guangzhou 510640, China

**Keywords:** rice, grain transparency, genome-wide association study, quantitative trait loci (QTL), candidate gene

## Abstract

Grain transparency (GT) is a key determinant of rice appearance quality and consumer preference, yet its genetic basis remains poorly understood. In this study, we evaluated GT in a panel of 450 rice accessions grown in two environments (Guangzhou and Yangjiang). Nineteen quantitative trait loci (QTL) for GT were identified by a genome-wide association study (GWAS), distributed across all rice chromosomes except chromosomes 2 and 12. Among these, four QTL (*qGT-1b*, *qGT-3b*, *qGT-4b*, and *qGT-5b*) were consistently detected in both environments. Notably, *qGT-3b* and *qGT-5b* co-localized with the grain shape genes *GRAIN SIZE 3* (*GS3*) and *GRAIN WIDTH 5* (*GW5*), respectively. For the novel and stable QTL *qGT-4b*, linkage disequilibrium decay analysis delimited an approximately 300 kb candidate region. Integrative transcriptomic and genomic analyses pointed to two candidate genes: *LOC_Os04g40370* (*OsFbx142*), encoding an F-box domain protein, and *LOC_Os04g40720* (*OsSUBSrP1*), encoding a subtilisin-like serine protease. Haplotype analysis further revealed that specific haplotypes of these genes were significantly associated with GT variation across environments. Our findings provide genetic insights into the regulation of rice grain transparency and offer candidate genes for further functional validation and potential application in improving rice appearance quality.

## 1. Introduction

Rice (*Oryza sativa* L.) is a cornerstone of global food security, serving as the primary staple for more than half of the world’s population. With rising living standards, consumer demand for high-quality rice has intensified, making the improvement of grain quality a primary objective in modern rice breeding programs. Rice grain quality is a multifaceted trait encompassing milling, cooking, and eating, nutritional, and appearance characteristics. Among these, appearance quality is one of the most immediate factors influencing consumer preference and market value, as it is the first attribute evaluated at the point of purchase [1].

Appearance quality is determined by several key traits, including grain size and shape, chalkiness, color, and transparency. Grain transparency (GT), which describes the translucency of the endosperm when light passes through it, is a particularly critical determinant of consumer acceptance. Transparent, vitreous grains are highly prized in the market, whereas opaque or dull grains are often perceived as being of lower quality [2]. The degree of transparency is primarily a function of the endosperm’s internal structure, specifically the arrangement and integrity of starch granules and the presence of air spaces within the grain. Opaque grains often exhibit starch granules with surface cavities or a loosely packed structure that scatters light, whereas transparent grains possess a densely packed, homogeneous endosperm with minimal light scattering [1]. Notably, this endosperm structure is highly sensitive to environmental factors; for example, high temperature stress during grain filling can disrupt carbon metabolism, interfere with proper starch granule assembly, increase intergranular spaces, and ultimately reduce transparency [3].

In addition to environmental cues, genetic factors are intrinsically involved in determining GT, as evidenced by the substantial variation among different rice varieties. However, the underlying genetic architecture remains largely unclear. Waxy rice exhibits poor transparency, with an endosperm that appears milky white and waxy. In contrast, non-waxy rice generally has better transparency, often appearing transparent or translucent. Significant differences in transparency are observed among various soft rice varieties; those with a cloudy appearance show poor transparency, whereas varieties with a good appearance exhibit relatively high transparency [4]. To date, only a limited number of QTL mapping studies have specifically targeted grain transparency as a primary trait. Jin et al. constructed a high-density genetic linkage map using genotyping-by-sequencing (GBS) in a population derived from a cross between Koshihikari and Nona Bokra, and identified four QTL of GT in an F_2_ population, designated *TP-1*, *TP-2*, *TP-3*, and *TP-4* [5].

Subsequent efforts to clone and characterize genes affecting GT have highlighted the central role of starch biosynthesis. The *Waxy* (*Wx*) gene, which encodes a granule-bound starch synthase (GBSS) responsible for amylose synthesis, is a major regulator. Different *Wx* alleles produce a range of amylose contents, which directly impact endosperm opacity, from the waxy, opaque phenotype of *wx* mutants to the translucent grains of varieties with moderate to high amylose [1]. Recent discoveries of novel alleles, such as *Wx^mw^*, which confers improved transparency despite low amylose content, demonstrate the potential for fine-tuning this trait [6]. Beyond *Wx*, other members of the starch synthase (SS) gene family, like *SSII-b*, have been implicated. Downregulation of *SSII-b* can result in transparent grains with very low amylose content, suggesting a complex interplay between different starch biosynthetic pathways [7]. Furthermore, a class of genes responsible for the floury endosperm (FLO) phenotype has been characterized. Mutations in genes like *FLO2* and *FLO4* (also known as *SSIIIa*) lead to a chalky or floury endosperm, demonstrating that disruptions in starch structure and the regulation of storage compound accumulation affect GT [8,9].

Despite these advances, the genetic variants underlying natural variation in GT remain largely unidentified. Genome-wide association study (GWAS) has emerged as a powerful approach for dissecting the genetic architecture of complex quantitative traits in rice, demonstrating high efficiency in functional gene mining. To date, GWAS has been extensively applied to investigate rice appearance quality, leading to the successful identification of numerous QTL associated with grain shape and chalkiness. However, GWAS specifically targeting GT remains scarce. Wang et al. [10] performed a GWAS for nine traits related to grain appearance and milling quality using 258 accessions from the 3K Rice Genome Project evaluated in two environments (Sanya and Shenzhen). For grain opacity, six QTL were detected on chromosomes 1, 2, 4, and 7, with five detected only in Sanya and one identified specifically in Shenzhen [10]. Although grain shape and chalkiness show strong phenotypic correlations with transparency, accumulating evidence suggests that GT is governed by genetic regulatory mechanisms distinct from those controlling grain shape and chalkiness.

In the present study, we performed GWAS for GT using 450 rice accessions across two environments. The GT of milled rice was evaluated using a professional appearance quality instrument and scored on a scale of 1 to 5, where grade 1 represents the highest transparency (vitreous endosperm) and grade 5 represents the lowest transparency (opaque or dull endosperm). Nineteen QTL were identified, with four stable QTL detected in both environments. *qGT-3b* and *qGT-5b* co-localized with *GRAIN SIZE 3* (*GS3*) and *GRAIN WIDTH 5* (*GW5*), respectively. For the novel QTL *qGT-4b*, integration of transcriptomic and genomic analyses revealed two candidate genes, *LOC_Os04g40370* (encoding an F-box domain-containing protein, *OsFbx142*) and *LOC_Os04g40720* (encoding a subtilisin-like serine protease, *OsSUBSrP1*). This study provides new insight into the genetic basis of GT in rice and contributes to molecular breeding for high-quality rice.

## 2. Results

### 2.1. Phenotypic Variation in GT in 450 Rice Accessions

**Figure 1:**
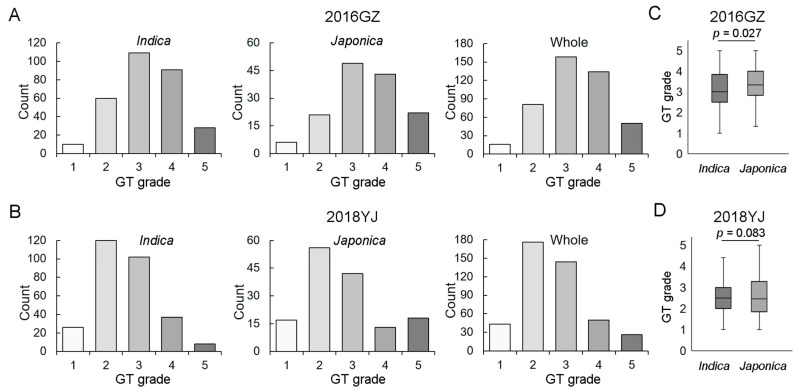
Phenotypic variation in grain transparency in 450 rice accessions across two environments. (**A**,**B**) Frequency distributions of GT grades in the *indica*, *japonica*, and whole populations in Guangzhou (2016GZ) and Yangjiang (2018YJ), respectively. The distributions approximate normality. (**C**,**D**) Comparison of GT between *indica* and *japonica* accessions in 2016GZ and 2018YJ. Data were analyzed using Student’s *t*-test for comparisons. Exact *p*-values are shown in the figure.

The GT of 450 rice accessions was evaluated in two environments (Guangzhou and Yangjiang). It is important to note that GT was scored on a scale of 1 to 5, where lower grades indicate higher transparency (vitreous endosperm) and better appearance quality. In both environments, the phenotypic distributions of GT in the *indica*, *japonica*, and whole populations were approximately normally distributed (Figure 1A,B), suggesting a relatively high level of diversity in the studied germplasm set. Furthermore, we observed that in the 2016GZ environment, *indica* accessions showed lower GT grades than *japonica* accessions (Figure 1C); however, in the 2018YJ environment, no significant difference in GT grades was detected between the two subspecies (Figure 1D). These results indicate that GT grades are susceptible to environmental influence.

### 2.2. QTL Mapping for GT by GWAS

**Figure 2:**
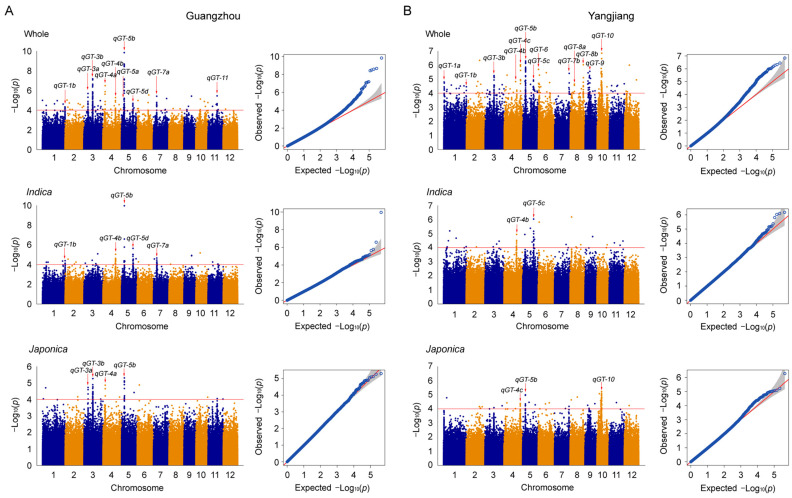
Genome-wide association study (GWAS) for grain transparency in 450 rice accessions. Manhattan plots for GT in (**A**) 2016GZ and (**B**) 2018YJ. The left and right panels represent the Manhattan scatter plot and the QQ-plot, respectively.

Using the GT phenotype data of 450 accessions from two ecological regions, a genome-wide association study (GWAS) was conducted based on the mixed linear model (MLM). A total of 19 QTL for GT were identified, which were distributed across all chromosomes except 2 and 12 (Figure 2; Table 1). Among these, only *qGT-1b*, *qGT-3b*, *qGT-4b*, and *qGT-5b* were consistently detected in both environments. Notably, *qGT-3b* and *qGT-5b* were co-localized with previously reported major grain shape genes *GS3* and *GW5* [11,12], respectively. Both loci exhibited strong genetic effects, suggesting that these two grain shape genes play important roles in regulating GT. *qGT-7a* was identified in the whole and *indica* populations in the 2016GZ environment and co-localized with the previously reported *qTr7.1* [10]. Interestingly, all the QTL were detectable in the whole population. In addition, several QTL were identified specifically in subpopulations: *qGT-1b*, *qGT-4b*, *qGT-5c*, *qGT-5d*, and *qGT-7a* were detected in the *indica* population, while *qGT-3a*, *qGT-3b*, *qGT-4a*, *qGT-4c*, and *qGT-10* were detected in the *japonica* population. *qGT-5b* was identified in the whole, *indica*, and *japonica* populations in 2016GZ, and in the whole and *japonica* populations in 2018YJ. These results indicate that the identified QTL are influenced not only by environmental factors but also by subpopulation differentiation.

### 2.3. Linkage Disequilibrium Decay Analysis of qGT-4b

**Figure 3:**
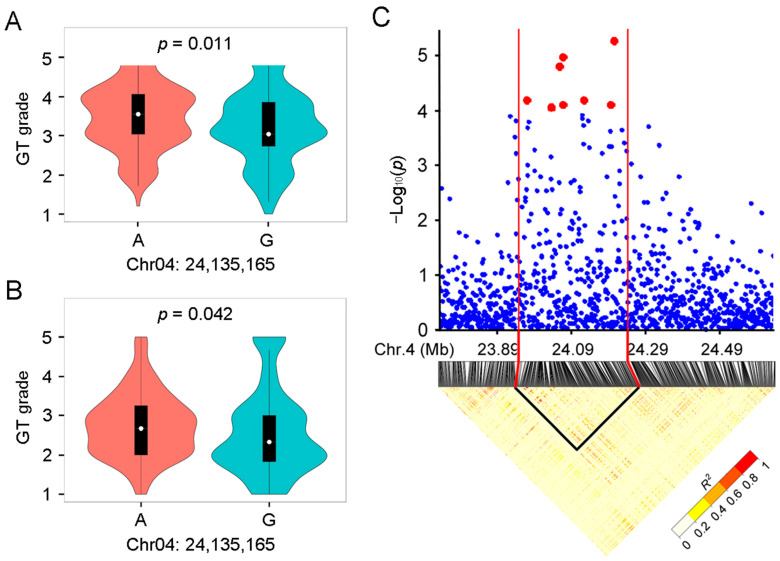
Linkage disequilibrium (LD) decay analysis of the *qGT-4b* locus. (**A**) Comparison in 2016GZ and (**B**) in 2018YJ of GT grades among different haplotypes defined by the most significant SNP within *qGT-4b*. Data were analyzed using Student’s *t*-test for comparisons. (**C**) LD decay plot across the *qGT-4b* region on chromosome 4. The region between the two red vertical lines represents the *qGT-4b* candidate interval (23.94–24.24 Mb). Red dots indicate SNPs with *p* value < 10^−4^.

Among the 19 QTL identified, *qGT-4b* stood out as a novel and stable locus: it was consistently detected in both environments across the whole and *indica* populations, and no GT-related QTL or gene has been previously reported in this region. We therefore focused on *qGT-4b* for subsequent candidate gene analysis. We compared the GT grades of accessions with different haplotypes based on the significant SNP in the *qGT-4b* region. Accessions carrying distinct haplotypes showed significantly different GT grades in both environments (Figure 3A,B). The linkage disequilibrium (LD) decay analysis in the *qGT-4b* region indicated that an approximately 300 kb interval (from 23.94 to 24.24 Mb on chromosome 4) was the putative region for this QTL (Figure 3C). A total of 53 annotated genes were located within this region (Table S2).

### 2.4. Candidate Gene Analysis of qGT-4b

**Figure 4:**
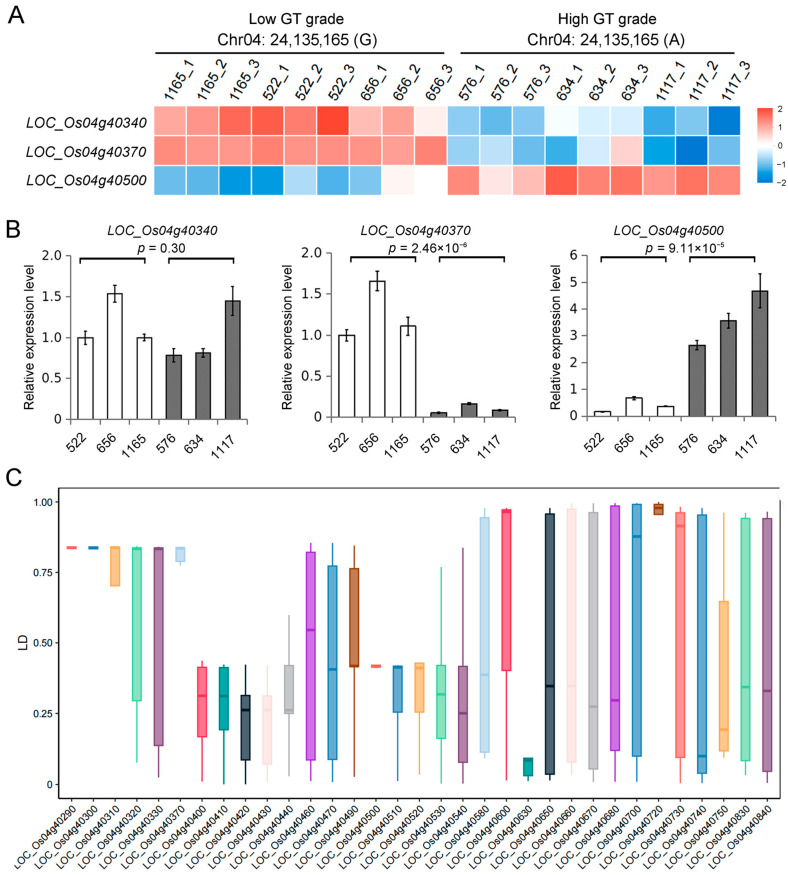
Candidate gene analysis of *qGT-4b* by transcriptomic and genomic approaches. (**A**) Heatmap of differentially expressed genes between low-GT grade and high-GT grade accessions based on RNA-seq data. (**B**) qRT-PCR validation of three selected genes (*LOC_Os04g40340*, *LOC_Os04g40370*, and *LOC_Os04g40500*). Error bars represent standard deviation (SD). Statistical comparisons were performed using a one-tailed Student’s *t*-test. (**C**) LD values of genes with the most significant SNP in *qGT-4b* region.

Based on haplotype analysis of *qGT-4b*, three accessions with low GT grades (i.e., lower numerical scores, indicating higher transparency and better quality) and three accessions with high GT grades (i.e., higher numerical scores, indicating lower transparency and poor quality) were selected for gene differential expression analysis. RNA-sequencing revealed that 19 genes were not expressed (defined as read count < 30, a threshold applied to exclude lowly expressed or spuriously mapped transcripts). Among the remaining 34 expressed genes, three genes (*LOC_Os04g40340*, *LOC_Os04g40370*, and *LOC_Os04g40500*) were differentially expressed between the two sets of contrasting lines (Figure 4A; Table S2). qRT-PCR assays confirmed the expression patterns of *LOC_Os04g40370* and *LOC_Os04g40500* measured by RNA-sequencing, but the results for *LOC_Os04g40340* were inconsistent with the RNA-sequencing data (Figure 4B). The two consistently validated genes exhibited opposite expression patterns: the expression levels of *LOC_Os04g40370* in accessions with low GT grades were significantly higher than those in accessions with high GT grades, whereas the expression levels of *LOC_Os04g40500* in accessions with low GT grades were significantly lower than those in accessions with high GT grades.

We further analyzed the correlation between each gene and the most significant SNP using linkage disequilibrium (LD). *LOC_Os04g40720* exhibited the highest LD value, indicating that at the genomic level, this gene showed the strongest association with GT. Among the two candidate genes obtained from transcriptome data, *LOC_Os04g40370* exhibited a relatively high LD value, whereas *LOC_Os04g40500* showed a low LD value (Figure 4C). Therefore, from the transcriptomic and genomic levels, the two most likely candidate genes for *qGT-4b* were identified: *LOC_Os04g40370* and *LOC_Os04g40720*.

### 2.5. Haplotype Analysis of Candidate Genes

**Figure 5:**
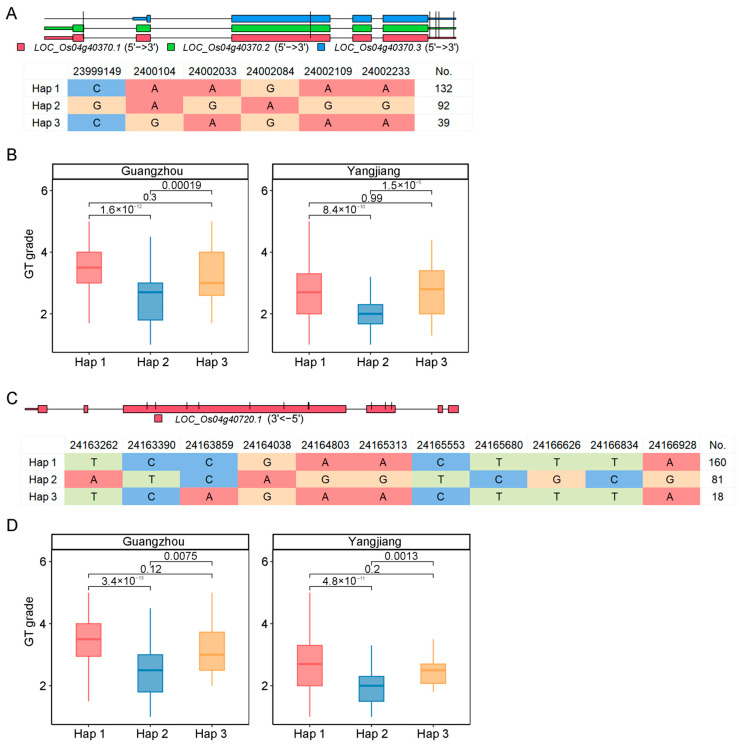
Haplotype analysis of the two candidate genes for *qGT-4b*. (**A**,**C**) Gene structures and variant positions of *LOC_Os04g40370* and *LOC_Os04g40720*, respectively. (**B**,**D**) Comparison of GT grades among different haplotypes of *LOC_Os04g40370* and *LOC_Os04g40720* in two environments. Data were analyzed using Student’s *t*-test for comparisons.

To explore the relationship between different haplotypes of the two candidate genes and GT grades, we analyzed variations in the promoter region (2000 bp upstream of the start codon), the 3’ untranslated region (3’ UTR), and the coding sequence (CDS). For *LOC_Os04g40370*, a total of six major variants were identified, two located in the CDS and four in the 3’ UTR (Figure 5A). For *LOC_Os04g40720*, 11 major variants were identified, all located in the CDS (Figure 5C). Based on these variants, three main haplotypes (Hap 1–3) were identified for both *LOC_Os04g40370* and *LOC_Os04g40720* (Figure 5A,C). Accessions carrying Hap 2 consistently exhibited lower GT grades than those carrying Hap 1 or Hap 3 in both environments, while no significant difference in GT grades was observed between Hap 1 and Hap 3 accessions in either environment (Figure 5B,D).

## 3. Discussion

### 3.1. Grain Transparency Is Environmentally Sensitive and Shares Genetic Determinants with Grain Shape

GT is an important indicator of rice appearance quality and represents a complex quantitative trait susceptible to environmental influence. Environmental factors such as high temperature stress can disrupt carbon metabolism in rice, interfere with starch structure formation, increase intergranular spaces, and ultimately reduce transparency [3]. Beyond direct effects on starch biosynthesis, heat stress is also known to trigger endoplasmic reticulum (ER) stress and the unfolded protein response (UPR) during grain filling, which disrupts the homeostasis of storage proteins and starch, compromises endosperm development, and promotes chalkiness formation [13,14]. This mechanistic insight provides a plausible molecular basis for the environmental instability of the GT QTL identified in our study and underscores the potential value of these loci for breeding heat-resilient rice varieties with stable appearance quality. In this study, we evaluated GT in 450 rice accessions across two ecological regions. The phenotypic trends of both *indica* and *japonica* populations varied between the two environments, further highlighting the role of environmental factors in determining grain transparency.

The molecular regulatory mechanisms underlying GT remain largely unclear, and relatively few QTL or genes have been identified directly targeting this trait, posing challenges for genetic improvement. In this study, a total of 19 QTL for GT were identified by GWAS. Among these, three QTL co-localized with previously reported loci: *qGT-7a* co-localized with *qTr7.1* [10], while *qGT-3b* and *qGT-5b* co-localized with the major grain shape genes *GS3* and *GW5* [11,12], respectively. These co-localization events suggest that these grain shape genes may regulate grain transparency primarily through morphology-mediated effects on endosperm development and starch packing, as grain filling proceeds from the outer to the inner endosperm and wider grains tend to exhibit compromised appearance quality [15,16]. However, we cannot exclude the possibility that these loci exert independent regulatory effects on endosperm development, and distinguishing between these mechanisms will require further investigation using near-isogenic lines. In addition to the two QTL co-localizing with grain shape genes, only two QTL (*qGT-1b* and *qGT-4b*) were consistently detected in both environments, further confirming that grain transparency is readily influenced by environmental conditions.

### 3.2. Grain Transparency and Chalkiness Are Partially Linked but Genetically Separable

Apart from grain shape and transparency, chalkiness is another important trait of rice appearance quality. Studies have shown that chalkiness affects transparency. Grains with high chalkiness or floury endosperm generally exhibit poor transparency [17], likely because a large proportion of loose tissue in the endosperm impairs light transmission. However, from the perspective of starch structure, chalkiness and transparency differ in the arrangement of starch granules; therefore, chalky grains do not necessarily show reduced transparency. Using the same panel of accessions, we previously identified QTL for chalkiness [18]. Some of those chalkiness QTL co-localized with transparency QTL identified in the present study, such as *qPGWC-1c^1wi^*/*qDEC-1^1wi^* with *qGT-1b*, and *qDEC-11c^1w^* with *qGT-11*. However, other transparency QTL, including *qGT-1a*, *qGT-4a*, *qGT-4b*, and *qGT-5a*, were located in different genomic regions. These results indicate that although grain transparency and chalkiness-related traits share closely linked genetic loci, and genetic determinants controlling transparency often also influence chalkiness, as both traits share a common structural foundation: starch granule packing and organization within the endosperm [4,19]. However, the structural basis of these two traits is not identical. Chalkiness primarily arises from loosely packed starch granules with intergranular voids, which scatter light and create opaque regions. In contrast, reduced transparency (dullness) is more specifically associated with the presence of cavities on the surface of starch granules and alterations in crystallinity and amylopectin chain-length distribution [20]. This structural distinction explains why some genetic loci affect both chalkiness and transparency (through shared effects on starch packing), while others influence only one of the two traits (through pathway-specific effects on granule surface structure or crystalline organization). Thus, although chalkiness and transparency share some genetic determinants, their partially distinct structural bases also allow for the identification of trait-specific QTL.

### 3.3. Two Candidate Genes at qGT-4b Are Implicated in Grain Transparency Regulation

Through combined transcriptomic and genomic analyses, two candidate genes for *qGT-4b* were identified. *LOC_Os04g40370* (*OsFbx142*) encodes an F-box domain-containing protein, and *LOC_Os04g40720* (*OsSUBSrP1*) encodes a putative subtilisin-like serine protease. The rice genome contains more than 779 F-box genes, which play diverse roles in regulating numerous physiological processes. Several studies have reported the involvement of F-box genes in regulating seed germination and grain quality. For instance, *OsFbx352* plays a regulatory role in glucose-induced suppression of seed germination by modulating abscisic acid metabolism [21]. Moreover, the *fbx365* mutant exhibits increased chalkiness and floury endosperm [22]. As an F-box domain-containing protein, OsFbx142 is predicted to function as a substrate-recognition subunit of the SCF (SKP1-CUL1-F-box) E3 ubiquitin ligase complex, targeting specific proteins for ubiquitin-dependent proteasomal degradation. However, no bona fide substrate of OsFbx142 has been identified to date, and its role in rice endosperm development has not been experimentally investigated. Given that several F-box proteins in rice have been reported to regulate grain development and seed germination [21,22], it is tempting to speculate that OsFbx142 may modulate the turnover of proteins involved in starch granule assembly or compound deposition during grain filling, thereby influencing endosperm compactness and transparency. Clearly, this hypothesis requires rigorous experimental validation. Future work, including yeast two-hybrid screening, co-immunoprecipitation coupled with mass spectrometry, and phenotypic characterization of *OsFbx142* knockout/overexpression lines, will be essential to uncover its authentic substrates and define its precise regulatory role in grain transparency.

Previous studies have shown that *OsSUBSrP1* is preferentially expressed in the upper part of young panicles. Knockout of *OsSUBSrP1* leads to defective anther cuticle formation, excessive water loss, and abnormal programmed cell death (PCD), ultimately causing spikelet abortion and reduced seed setting [23]. Although *OsSUBSrP1* has not been directly linked to grain transparency before, several lines of evidence suggest its potential involvement in this trait. First, grain transparency is largely determined by the physical structure of the endosperm, including the arrangement of starch granules and the presence of protein bodies or air spaces. Lipid metabolism, particularly the synthesis of cutin and wax, has been shown to influence grain filling and endosperm texture in rice. The downregulation of wax and cutin biosynthesis genes observed in the *OsSUBSrP1* mutant may therefore affect the deposition of storage compounds in developing grains, thereby altering transparency. Second, the expression of *OsSUBSrP1* in young panicles implies a role during early reproductive development, which could have cascading effects on grain quality traits, including transparency. Third, PCD in developing grain endosperm is tightly coupled with starch accumulation, and excessive or untimely PCD has been shown to compromise grain quality by limiting storage compound deposition [16]. Taken together, we propose that *OsSUBSrP1* may regulate grain transparency by modulating lipid metabolism-related processes or through PCD-related pathways during grain development. However, the mechanistic connection between its biochemical activity as a subtilisin-like serine protease and the structural organization of starch granules in the endosperm remains to be established. Future studies are needed to identify the downstream targets of OsSUBSrP1 and to determine whether its protease activity directly or indirectly influences starch granule packing or air space formation in the endosperm. Furthermore, given that endosperm development involves spatially distinct cell layers with specialized functions in starch accumulation, and that starch granule packing ultimately determines grain transparency, bulk RNA-seq inevitably averages over cell-type-specific transcriptional heterogeneity. Future studies employing single-cell RNA sequencing and spatial transcriptomics would enable high-resolution dissection of gene expression dynamics across different endosperm cell types, potentially revealing cell-layer-specific regulatory mechanisms underlying transparency.

## 4. Materials and Methods

### 4.1. Plant Materials

The 450 rice accessions (Table S1) were used for phenotype evaluation and GWAS in this study. These rice accessions were selected from the Rice Diversity Panel 2 (RDP2) [24] based on their origins and diversity, including 300 *indica* and 150 *japonica*. The sequencing data of these 450 rice accessions have been published in previous studies [25]. The 450 rice accessions were planted in two environments: Guangzhou (2016GZ) and Yangjiang (2018YJ) in Guangdong Province, China, during the second cropping seasons of 2016 and 2018, respectively. The experiments were arranged in a randomized complete block design, with each accession planted in two rows (as within-field replicates) per block. Field management, including irrigation, fertilization, and pest and disease control, followed standard local agronomic practices for rice production in Guangdong Province. Routine water and fertilizer regimes were applied according to local conventions, and no experimental treatments were imposed.

### 4.2. Phenotypic Evaluation of GT

At full maturity, grains were harvested, naturally dried, and then stored at 15 °C. For each accession, 20 g of paddy rice was de-husked using a huller (JLG-III, Chengdu, Sichuan, China) and milled using a polisher (JNM-III, Chengdu, Sichuan, China). The grain transparency (GT) of head rice was measured using a Rice Appearance Quality Determination Instrument (SC-E, Hangzhou, Zhejiang, China). The measured GT values were classified into five grades ranging from 1 to 5, where grade 1 denotes high GT and good grain quality, and grade 5 denotes low GT and poor grain quality. All measurements were performed on three independent subsamples of 20 g paddy rice per accession, and the average grade was used for subsequent GWAS.

### 4.3. GWAS and QTL Delimitation

GWAS was conducted using GAPIT version 2 software, following the procedures described in our previous study [18]. Briefly, single-nucleotide polymorphisms (SNPs) were filtered based on the following criteria: missing data rate < 30% and minor allele frequency (MAF) > 0.05. A mixed linear model (MLM) incorporating a kinship matrix was applied for GWAS, with the number of principal components set to 3 in GAPIT. Manhattan and QQ plots were generated using the R package qqman (version 0.1.9). A genomic region harboring two or more significant SNPs (*p* < 0.0001) within a 200 kb interval was considered a single QTL.

### 4.4. Linkage Disequilibrium (LD) Decay Analysis

The SNP information of 800 kb (23.74–24.54 Mb) for *qGT-4b* was used for LD decay analysis. The LD heatmap was generated following the methods described in previous study [26]. To identify candidate genes at the *qGT-4b* locus using linkage disequilibrium (LD) decay analysis, the most significant SNP within the *qGT-4b* interval from the GWAS was first determined. For each gene located in this interval, all SNPs within the gene itself, as well as within 2000 bp upstream and downstream of the gene, were extracted to form a SNP set. The LD between each of these SNPs and the most significant SNP in the *qGT-4b* interval was then calculated. This procedure was repeated for every gene in the *qGT-4b* region. In principle, a higher LD value indicates a stronger linkage between the SNPs associated with a given gene and the most significant SNP in the QTL interval, suggesting that the gene is more likely to be involved in the trait variation.

### 4.5. RNA-Sequencing

Based on haplotype analysis of *qGT-4b*, six accessions (three with low GT grade and three with high GT grade) with similar flowering time were selected for RNA-seq. For each accession, three independent biological replicates (developing panicles at 15 days after flowering) were sampled, and total RNA was extracted individually from each replicate using Trizol reagent (Invitrogen, Carlsbad, CA, USA). RNA-sequencing was conducted by Annoroad Gene Technology (Beijing, China). Sequencing libraries with 150 bp paired-end reads were constructed and sequenced on the Illumina HiSeq X Ten platform (Illumina, San Diego, CA, USA). Raw reads were processed using fastp for quality trimming. All subsequent data analyses followed the procedures described in our previous study [26]. Briefly, raw reads were processed for quality trimming, and clean reads were aligned to the Nipponbare reference genome (MSU v7.0). Only uniquely mapped reads were retained for gene read counting, and differential expression analysis between accessions with low GT grade and high GT grade was performed with fold change thresholds of ≥2 or ≤0.5 and an adjusted *p*-value < 0.01.

### 4.6. Real-Time PCR Analysis

The same RNA samples used for RNA-sequencing were used to validate the expression levels of candidate genes. Equal amounts of RNA from the three biological replicates of each accession were pooled. The cDNA synthesis was conducted using the PrimeScript^TM^ RT reagent kit (Takara, Kusatsu, Shiga, Japan). qRT-PCR analysis was carried out using the Bio-Rad CFX 96 system (Bio-Rad, Hercules, CA, USA). The reaction mixture (20 μL) contained 10 μL of SYBR Green Master Mix (2×), 0.5 μL each of forward and reverse primers (10 μM), and 2 μL of diluted cDNA, and ddH_2_O was added to bring the final volume to 20 μL. The thermal cycling program was: 95 °C for 3 min, followed by 40 cycles of 95 °C for 10 s and 60 °C for 30 s, with a melt curve analysis from 65 °C to 95 °C. Primers were designed using the NCBI Primer Designing Tool, and the gene-specific primer sequences are listed in Supplementary Table S3. The rice EF1α gene was used as an internal control, as it has been validated as a stable reference gene for qRT-PCR in rice [27]. Relative expression levels were calculated using the 2^−ΔΔCt^ method. All reactions were performed in triplicate.

## Figures and Tables

**Table 1:** QTL identified for GT by GWAS.

QTL	Chromosome	Environment	Population	Position (bp)	*p*-Value	Co-Located QTL or Cloned Gene
*qGT-1a*	1	Yangjiang	Whole	752,096	1.60 × 10^−5^	
*qGT-1b*	1	Guangzhou	Whole *Indica*	43,080,302	4.91 × 10^−5^	
		Yangjiang	Whole	43,177,205	4.05 × 10^−5^	
*qGT-3a*	3	Guangzhou	Whole *Japonica*	7,876,802	1.09 × 10^−6^	
*qGT-3b*	3	Guangzhou	Whole *Japonica*	16,869,049	6.21 × 10^−8^	[11]
		Yangjiang	Whole	16,878,653	5.55 × 10^−6^	
*qGT-4a*	4	Guangzhou	Whole *Japonica*	4,293,668	9.38 × 10^−8^	
*qGT-4b*	4	Guangzhou	Whole *Indica*	24,135,165	8.14 × 10^−6^	
		Yangjiang	Whole *Indica*	24,024,612	7.07 × 10^−5^	
*qGT-4c*	4	Yangjiang	Whole *Japonica*	31,029,056	8.9 × 10^−7^	
*qGT-5a*	5	Guangzhou	Whole	4,298,919	5.73 × 10^−5^	
*qGT-5b*	5	Guangzhou	Whole *Indica* *Japonica*	5,359,520	1.79 × 10^−10^	[12]
		Yangjiang	Whole *Japonica*	5,366,703	5.03 × 10^−7^	
*qGT-5c*	5	Yangjiang	Whole *Indica*	21,167,792	4.93 × 10^−5^	
*qGT-5d*	5	Guangzhou	Whole *Indica*	21,924,400	2.22 × 10^−5^	
*qGT-6*	6	Yangjiang	Whole	864,175	1.8 × 10^−5^	
*qGT-7a*	7	Guangzhou	Whole *Indica*	6,281,713	2.44 × 10^−6^	[10]
*qGT-7b*	7	Yangjiang	Whole	27,553,310	2.68 × 10^−6^	
*qGT-8a*	8	Yangjiang	Whole	7,796,155	2.28 × 10^−5^	
*qGT-8b*	8	Yangjiang	Whole	24,785,271	8.05 × 10^−5^	
*qGT-9*	9	Yangjiang	Whole	7,993,424	6.88 × 10^−6^	
*qGT-10*	10	Yangjiang	Whole *Japonica*	8,720,459	1.45 × 10^−7^	
*qGT-11*	11	Guangzhou	Whole	17,446,307	3.72 × 10^−6^	

## Data Availability

The original contributions presented in this study are included in the article and its Supplementary Materials. Further inquiries can be directed to the corresponding authors.

## Supplementary Materials

The following supporting information can be downloaded at: https://www.mdpi.com/article/10.3390/ijms27135835/s1.

## References

[1] Zhao D.S., Zhang C.Q., Li Q.F., Liu Q.Q. (2022). Genetic control of grain appearance quality in rice. Biotechnol. Adv..

[2] Ren D.Y., Ding C.Q., Qian Q. (2023). Molecular bases of rice grain size and quality for optimized productivity. Sci. Bull..

[3] Liu Z.C., Xia S.M., Li Y.X., Li H.B., Zhu M.H., Yin H.R., Wang Z.M., Man J.G., Xiong D.L., Cui K.H. (2026). Post-heading high nighttime temperature impairs grain protein-starch balance and rice quality through altering nitrogen metabolism. Plant Cell Environ..

[4] Fan P., Xu J., Wang Z.J., Liu G.D., Zhang Z.Z., Tian J.Y., Wei H.Y., Zhang H.C. (2023). Phenotypic differences in the appearance of soft rice and its endosperm structural basis. Front. Plant Sci..

[5] Jin S.K., Xu L.N., Yang Q.Q., Zhang M.Q., Wang S.L., Wang R.A., Tao T., Hong L.M., Guo Q.Q., Jia S.W. (2023). High-resolution quantitative trait locus mapping for rice grain quality traits using genotyping by sequencing. Front. Plant Sci..

[6] Zhang C.Q., Yang Y., Chen S.J., Liu X.J., Zhu J.H., Zhou L.H., Lu Y., Li Q.F., Fan X.L., Tang S.Z. (2021). A rare *Waxy* allele coordinately improves rice eating and cooking quality and grain transparency. J. Integr. Plant Biol..

[7] Li Q.F., Huang L.C., Chu R., Li J., Jiang M.Y., Zhang C.Q., Fan X.L., Yu H.X., Gu M.H., Liu Q.Q. (2018). Down-regulation of *SSSII-2* gene expression results in novel low-amylose rice with soft, transparent grains. J. Agric. Food Chem..

[8] Fujita N., Yoshida M., Kondo T., Saito K., Utsumi Y., Tokunaga T., Nishi A., Satoh H., Park J.H., Jane J.L. (2007). Characterization of *SSIIIa*-deficient mutants of rice: The function of *SSIIIa* and pleiotropic effects by *SSIIIa* deficiency in the rice endosperm. Plant Physiol..

[9] She K.C., Kusano H., Koizumi K., Yamakawa H., Hakata M., Imamura T., Fukuda M., Naito N., Tsurumaki Y., Yaeshima M. (2010). A novel factor FLOURY ENDOSPERM2 is involved in regulation of rice grain size and starch quality. Plant Cell.

[10] Wang X.Q., Pang Y.L., Wang C.C., Chen K., Zhu Y.J., Shen C.C., Ali J., Xu J.L., Li Z.K. (2017). New candidate genes affecting rice grain appearance and milling quality detected by genome-wide and gene-based association analyses. Front. Plant Sci..

[11] Mao H.L., Sun S.Y., Yao J.L., Wang C.R., Yu C.G., Li X.H., Zhang Q.F. (2010). Linking differential domain functions of the GS3 protein to natural variation of grain size in rice. Proc. Natl. Acad. Sci. USA.

[12] Weng J., Gu S., Wan X.Y., Gao H., Guo T., Su N., Lei C.L., Zhang X., Cheng Z.J., Guo X.P. (2008). Isolation and initial characterization of *GW5*, a major QTL associated with rice grain width and weight. Cell Res..

[13] Ali M., Ma X., Ali I., Hu S. (2025). Native genetic switch enhances heat resilience, grain quality, and yield in rice. J. Integr. Plant Biol..

[14] Li W., Yang K., Hu C.F., Abbas W., Zhang J., Xu P.K., Cheng B., Zhang J.C., Yin W.J., Shalmani A. (2025). A natural gene on-off system confers field thermotolerance for grain quality and yield in rice. Cell.

[15] Ma B., Zhang L., He Z.H. (2023). Understanding the regulation of cereal grain filling: The way forward. J. Integr. Plant Biol..

[16] Chen L., Li X.M., Zheng M.H., Hu R., Dong J.F., Zhou L.Y., Liu W.G., Liu D.L., Yang W. (2024). Genes controlling grain chalkiness in rice. Crop J..

[17] Dong H., Lei J., Tian Y.L., Liu J., Yang H., Jiang X.K., Zhang R.S., Zhang Y., Chen R.B., Bao Y.Q. (2026). D-amino acid aminotransferase1 regulates grain chalkiness in rice by modulating endoplasmic reticulum stress response. Proc. Natl. Acad. Sci. USA.

[18] Huo X., Wang J., Chen L., Fu H., Yang T.F., Dong J.F., Ma Y.M., Zhou L., Chen J.S., Liu D.L. (2023). Genome-wide association mapping and gene expression analysis reveal candidate genes for grain chalkiness in rice. Front. Plant Sci..

[19] Ying Y.N., Hu Y.Q., Liu X.X., Zhao J.J., Deng B.W., Zhang Z.W., Bao J.S. (2025). Effects of *Wx*, *SSIIa* and *FLO2* alleles and their interactions on the formation of multi-scale structures of rice starch. Int. J. Biol. Macromol..

[20] Fan P., Wang W.T., Xu J., Xu F.F., Li G.Y., Wei H.Y., Zhang H.C., Liu G.D. (2024). Starch-related structural basis and enzymatic mechanism of the different appearances of soft rice. Int. J. Biol. Macromol..

[21] Song S.Y., Dai X.Y., Zhang W.H. (2012). A rice F-box gene, *OsFbx352*, is involved in glucose-delayed seed germination in rice. J. Exp. Bot..

[22] Yuan J.Y., Chen S.S., Jiao W., Wang L.F., Wang L.M., Ye W.X., Lu J., Hong D.L., You S.L., Cheng Z.K. (2017). Both maternally and paternally imprinted genes regulate seed development in rice. New Phytol..

[23] Ali A., Wu T.K., Zhang H.Y., Xu P.Z., Zafar S.A., Liao Y.X., Chen X.Q., Zhou H., Liu Y.T., Wang W.M. (2022). A putative *SUBTILISIN-LIKE SERINE PROTEASE 1* (*SUBSrP1*) regulates anther cuticle biosynthesis and panicle development in rice. J. Adv. Res..

[24] McCouch S.R., Wright M.H., Tung C.W., Maron L.G., McNally K.L., Fitzgerald M., Singh N., DeClerck G., Agosto-Perez F., Korniliev P. (2016). Open access resources for genome-wide association mapping in rice. Nat. Commun..

[25] Wang J., Yang W., Zhang S.H., Hu H.F., Yuan Y.X., Dong J.F., Chen L., Ma Y.M., Yang T.F., Zhou L. (2023). A pangenome analysis pipeline provides insights into functional gene identification in rice. Genome Biol..

[26] Nie S., Chen L., Zheng M.H., Dong J.F., Ma Y.M., Zhou L., Wang J., Chen J.S., Hu H.F., Yang T.F. (2024). GWAS and transcriptomic analysis identify *OsRING315* as a new candidate gene controlling amylose content and gel consistency in rice. Rice.

[27] Jain M., Nijhawan A., Tyagi A.K., Khurana J.P. (2006). Validation of housekeeping genes as internal control for studying gene expression in rice by quantitative real-time PCR. Biochem. Biophys. Res. Commun..

